# Neurological and Olfactory Disturbances After General Anesthesia

**DOI:** 10.3390/life15030344

**Published:** 2025-02-22

**Authors:** Antonino Maniaci, Mario Lentini, Rosario Trombadore, Loris Gruppuso, Santo Milardi, Rosario Scrofani, Giuseppe Cuttone, Massimiliano Sorbello, Rodolfo Modica, Jerome R. Lechien, Paolo Boscolo-Rizzo, Daniele Salvatore Paternò, Luigi La Via

**Affiliations:** 1 Deparment of Medicine and Surgery, University of Enna “Kore”, 94019 Enna, Italy; mario.lentini@unikore.it (M.L.); giuseppe.cuttone@unikore.it (G.C.); massimiliano.sorbello@unikore.it (M.S.); 2 Asp 7 Ragusa, 97100 Ragusa, Italy; rosario.trombadore@asp.rg.it (R.T.); loris.gruppuso@asp.rg.it (L.G.); santo.milardi@asp.rg.it (S.M.); rosario.scrofani@asp.rg.it (R.S.); rodolfo.modica@asp.rg.it (R.M.); paternomd@icloud.com (D.S.P.); 3 Division of Laryngology and Bronchoesophagology, Department of Otolaryngology Head Neck Surgery, EpiCURA Hospital, UMONS Research Institute for Health Sciences and Technology, Faculty of Medicine, University of Mons (UMons), 7000 Mons, Belgium; jerome.lechien@umons.ac.be; 4 Department of Medical, Surgical and Health Sciences, Section of Otolaryngology, University of Trieste, 34121 Trieste, Italy; paolo.boscolorizzo@units.it; 5 Department of Anesthesia and Intensive Care, University Hospital Policlinico “G. Rodolico-San Marco”, 95123 Catania, Italy; luigilavia7@gmail.com

**Keywords:** general anesthesia, neurological disturbances, olfactory dysfunction, postoperative cognitive dysfunction (POCD), neuroinflammation, oxidative stress, olfactory bulb, postoperative complications, cognitive recovery, biomarkers

## Abstract

Neurological and olfactory disturbances are increasingly recognized as potential complications of general anesthesia, particularly in vulnerable populations, such as the elderly, children, and individuals with comorbidities. Recent studies have highlighted the need for tailored anesthetic approaches in these high-risk groups to mitigate potential long-term effects. These disturbances, including postoperative cognitive dysfunction, delirium, and olfactory deficits, often arise from shared pathophysiological mechanisms, such as neuroinflammation, oxidative stress, and disruptions in cerebral perfusion. The olfactory system is particularly susceptible to anesthesia-induced neurotoxicity given its proximity to central nervous system structures and its role in sensory and cognitive processing. Furthermore, the unique regenerative capacity of olfactory neurons may be compromised by prolonged or repeated exposure to anesthetic agents, potentially leading to long-term olfactory dysfunction. Risk factors, such as advanced age, neurodegenerative diseases, diabetes, cardiovascular conditions, genetic predispositions, and the type and duration of anesthesia exposure, further exacerbate these complications. Preventive strategies, including comprehensive preoperative risk assessment, personalized anesthetic protocols based on genetic and physiological profiles, and proactive postoperative care with early intervention programs, are critical for reducing impairments and improving long-term patient outcomes. Emerging evidence highlights the potential role of neuroprotective agents, such as antioxidants and anti-inflammatory therapies, in mitigating the effects of anesthesia-induced neurotoxicity. Longitudinal studies are needed to evaluate the long-term effects of anesthesia on cognitive and sensory health, particularly in high-risk populations. These studies should incorporate advanced neuroimaging techniques and biomarker analysis to elucidate the underlying mechanisms of anesthesia-induced neurological and olfactory disturbances. This narrative review provides a comprehensive overview of the mechanisms, risk factors, and preventive strategies for neurological and olfactory disturbances after general anesthesia and highlights future directions for research to improve patient outcomes. We conducted a comprehensive literature search using databases, such as PubMed and Scopus, to identify relevant studies.

## 1. Introduction

General anesthesia (GA) is an essential component of modern medicine, enabling the safe performance of complex surgeries by inducing reversible unconsciousness, analgesia, amnesia, and muscle relaxation [1,2,3]. Despite its invaluable role in surgical practice, growing evidence suggests that GA can have lasting effects on the nervous system, particularly in vulnerable populations, such as the elderly, children, and individuals with pre-existing neurological conditions [3,4,5,6,7]. Among these effects, postoperative cognitive dysfunction (POCD) and other neurological impairments have been well-documented [1,2,3,4]. Recently, increasing attention has been directed toward the relationship between GA and olfactory dysfunction, as the olfactory system is uniquely vulnerable to both systemic and neurological insults. This review explores the potential links between neurological and olfactory disorders following GA, with a focus on shared mechanisms, risk factors, and clinical implications. The olfactory system plays a critical role in both sensory and neurological functions. It is one of the few sensory systems with direct access to the central nervous system, as the olfactory bulb connects to the brain’s limbic and cortical areas without synapsing in the thalamus. This close integration with cognitive and emotional centers, including the hippocampus and the orbitofrontal cortex, underscores its susceptibility to neurological changes [3,4]. Additionally, the olfactory system’s regenerative capacity, through continuous turnover of olfactory receptor neurons, makes it particularly sensitive to disruptions caused by inflammation, neurotoxicity, or ischemia, all of which can be exacerbated by anesthetic agents [5]. Understanding the interplay between GA, olfactory dysfunction, and broader neurological disorders is essential for identifying at-risk patients and improving anesthetic protocols. Postoperative cognitive dysfunction (POCD) and delirium are among the most studied complications of GA. POCD presents as a transient or organ weakness of memory, attention, and executive function, especially in older patients [6]. Research indicates that these cognitive changes can last for several weeks to months after surgery, significantly affecting quality of life and functional independence [7]. Postoperative delirium displayed significant associations with neuroinflammation as well as neurophysiological derangements from cerebral perfusion changes due to anesthesia and surgical stress [8]. A gap in the literature has now been fulfilled to focus on the bond between olfactory dysfunction and GA. The olfactory system is a key player in sensory and neurological function, with a direct link to the central nervous system on its own. Its intimate interface with cognitive and emotional centers, including the hippocampus and the orbitofrontal cortex, emphasizes its vulnerability to neurologic change [3]. Moreover, the continuous turnover of olfactory receptor neurons reflects the regenerative capacity of the olfactory system, making its development vulnerable to disturbances that can be exacerbated by anesthetic agents, such as inflammation, neurotoxicity, or ischemia [5]. New data indicate that GA disrupts olfaction through direct and indirect pathways. Other anesthetic agents, such as isoflurane and sevoflurane, have also induced neuroinflammation and generated oxidative stress extending to damage of the olfactory epithelium or the olfactory bulb [9,10]. Other systemic effects of anesthesia (e.g., hypoxia, altered blood flow) can damage the delicate vasculature of the olfactory system. Patients with general anesthesia (GA) for major surgeries have reduced olfaction immediately after surgery and even for weeks, according to a recent study [10,11]. The connection between olfactory dysfunction and broader neurological disorders is of particular interest. While the prevalence of long-term olfactory dysfunction after GA remains unclear, these findings raise concerns about the potential underreporting of this complication in clinical practice. The connection between olfactory dysfunction and neurological disorders extends beyond the immediate effects of anesthesia. Olfactory impairment is increasingly recognized as an early biomarker of neurodegenerative diseases, such as Parkinson’s disease and Alzheimer’s disease [12,13]. Given the shared mechanisms of neuroinflammation, oxidative stress, and synaptic damage observed in both neurodegenerative conditions and anesthesia-induced neurological changes, it is plausible that olfactory dysfunction after GA may signal broader disruptions in brain health. For example, research has shown that anesthetic agents can affect amyloid-beta processing, a hallmark of Alzheimer’s pathology, which may exacerbate cognitive decline and sensory dysfunction in predisposed individuals [14]. Similarly, olfactory dysfunction has been linked to hippocampal atrophy, a key feature of cognitive impairment, suggesting a potential overlap in the pathways affected by anesthesia and neurodegeneration [15]. Risk factors for both olfactory and neurological disorders following GA include advanced age, pre-existing cognitive or sensory deficits, and comorbidities, such as diabetes and cardiovascular disease. Risk factors for both olfactory and neurological disorders following GA include advanced age, pre-existing cognitive or sensory deficits, and comorbidities, such as diabetes and cardiovascular disease [16]. Elderly patients, in particular, are more susceptible to the neurotoxic effects of anesthetic agents due to reduced neuronal plasticity and the accumulation of chronic inflammation [16]. Pediatric populations, while less studied, may also face risks to their developing nervous systems, with some studies highlighting long-term cognitive and sensory effects in children exposed to repeated anesthesia [17]. Identifying these at-risk populations is critical for tailoring perioperative care and minimizing complications. This narrative review analyzed neurological and olfactory disorders as potential related complications of general anesthesia, particularly in predisposed populations, such as the elderly, children, and individuals with comorbidities.

## 2. Review Methodology

This narrative review provides a synthesis of the literature regarding neurological and olfactory disturbances following general anesthesia, with emphasis on elucidating their mechanisms, risk factors, and clinical impact. Peer-reviewed studies on this matter were identified through a literature search using electronic databases, such as PubMed, Scopus, Web of Science, and Google Scholar.

The PICO framework was used to inform this review. The Population (P) was patients who received general anesthesia for surgical operations. The Intervention (I) was exposure to general anesthetic agents, including volatile anesthetics (isoflurane, sevoflurane) and intravenous agents (propofol, dexmedetomidine). The Comparison (C) was patients without general anesthesia or those under regional/local anesthetic. Outcome (O): Neurological complications, including postoperative cognitive dysfunction (POCD), delirium, and long-term cognitive decline, as well as olfactory dysfunction, including hyposmia, anosmia, or olfactory bulb damage. The search strategy included the medical subject heading (MeSH) terms as well as free-text search terms, such as general anesthesia, neurological dysfunction, olfactory dysfunction, postoperative cognitive dysfunction (POCD), neuroinflammation, oxidative stress, and cognitive recovery postoperatively. No time restriction was present; all studies written in English were included to ensure a broad review of the latest research. Specific inclusion and exclusion criteria were used to ensure the relevance of studies. Peer-reviewed research articles, systematic reviews, meta-analyses, and clinical trials were included if they investigated how general anesthesia may affect neurological or olfactory functions. Substantial specific relevance was found in studies examining underlying pathophysiological mechanisms, risk factors, and preventive strategies. Animal and human studies were included in order to encompass the range of evidence supporting the neurotoxic effects of anesthetic agents. Conversely, when studies referred only to regional or local anesthesia and no mention was made of general anesthesia, these were excluded; case reports, opinion pieces, and editorials were excluded unless novel contributions were made. Studies addressing other non-anesthesia-related causes of neurological or olfactory dysfunction, such as primary neurodegenerative disorders, were not included in order to remain focused on anesthetic-induced effects. The selected studies were subjected to a qualitative synthesis organized by major topics, which included (i) neurological complications of general anesthesia, (ii) olfactory disturbances post anesthesia exposures, (iii) common mechanisms associated with both complications, and (iv) prevention strategies. Postoperative cognitive dysfunction and delirium as neurological complications were reviewed with respect to neuroinflammation and synaptic and vascular changes. The preponderance of evidence implicates anesthesia in damaging the olfactory system, including the olfactory bulb, and ruining receptor neuron regeneration. Studies exploring oxidative stress, neuroinflammatory pathways, and changes in cerebral perfusion were examined to reveal overlapping mechanisms that may underlie both the dysregulation of sensory processing and cognitive impairment. In addition, the literature pertaining to patient-centric risk factors and perioperative alterations was evaluated to determine whether any methods for ameliorating such effects exist. No formal meta-analysis was performed for this review due to substantial heterogeneity between the study designs, outcome measures, and methodologies, which hindered the possibility of a quantitative synthesis. A narrative-style approach was taken to synthesize existing knowledge and highlight important research opportunities.

## 3. General Anesthesia: Mechanisms and Neurological Implications

GA works by inducing a state of reversible unconsciousness through the modulation of neural activity, primarily in the central nervous system (CNS). Its effects are achieved via interactions with specific molecular targets, including gamma-aminobutyric acid (GABA) receptors, N-methyl-D-aspartate (NMDA) receptors, and potassium channels [18]. While these mechanisms allow for the suppression of sensory input, pain perception, and motor reflexes, they can also lead to unintended neurological consequences, both during and after the anesthetic process. Research has increasingly linked GA to postoperative neurological impairments, including POCD, delirium, and even long-term neurodegeneration, particularly in vulnerable populations [19,20]. One of the primary concerns with GA is its potential to disrupt neural homeostasis through neuroinflammation and oxidative stress. Anesthetic agents, such as isoflurane, sevoflurane, and propofol, have been shown to activate microglial cells, triggering the release of pro-inflammatory cytokines, such as tumor necrosis factor-alpha (TNF-α) and interleukin-6 (IL-6) [21]. This inflammatory response, while initially aimed at protecting neural tissue, can lead to synaptic dysfunction and neuronal damage if prolonged. For example, animal studies have demonstrated that exposure to isoflurane increases levels of reactive oxygen species (ROS) in the hippocampus, a brain region critical for memory and learning, potentially contributing to cognitive deficits [22]. These findings suggest that the neurological effects of GA are not limited to the duration of surgery but may extend into the postoperative period. Another significant mechanism through which GA affects the nervous system is through alterations in cerebral blood flow and oxygenation. GA can cause dose-dependent reductions in cerebral metabolic rate and blood flow, which may lead to hypoperfusion and ischemia in certain brain regions [23]. This is particularly concerning in older adults, who already experience age-related declines in vascular function and cerebral autoregulation. Studies have shown that patients with pre-existing cerebrovascular disease or other comorbidities are at an elevated risk of postoperative neurological complications, including strokes and cognitive decline [24]. Moreover, disturbances in cerebral perfusion can also impair the olfactory bulb and related cortical areas, which are highly dependent on adequate oxygenation for normal function [25]. The relationship between GA and postoperative delirium further highlights the impact of anesthesia on the brain. Evidence suggests that GA contributes to delirium through mechanisms like cholinergic dysfunction, neuroinflammation, and disruptions in circadian rhythms [26]. These changes may persist beyond the acute phase, with some studies linking postoperative delirium to an increased risk of long-term cognitive decline and dementia [27]. The overlap between delirium and olfactory dysfunction is an area of growing interest, as both conditions may share common pathways of neuroinflammation and synaptic disruption. Interestingly, not all neurological effects of GA are exclusively negative. Some anesthetic agents, such as propofol, have shown neuroprotective properties in certain contexts, particularly in reducing excitotoxicity and preserving blood–brain barrier integrity during ischemic events [28]. However, the balance between protective and harmful effects depends on factors like the dosage, the duration of exposure, and patient-specific vulnerabilities. For instance, while low doses of propofol may reduce oxidative stress, prolonged or excessive exposure to anesthetics has been associated with increased neurotoxicity [29]. This duality underscores the complexity of GA’s impact on the CNS and highlights the need for individualized anesthetic management. The effects of GA on the nervous system are not limited to adults. Pediatric populations are also at risk, as the developing brain is highly sensitive to disruptions in neural activity. Studies in animal models have shown that exposure to GA during critical periods of brain development can lead to widespread neuronal apoptosis, particularly in regions like the prefrontal cortex and the hippocampus, which are essential for cognitive function [30]. Clinical studies have also raised concerns about potential long-term effects on neurodevelopment in children exposed to repeated or prolonged anesthesia, with some reporting deficits in memory, attention, and language skills [31]. These findings have prompted ongoing research into the safety of anesthetic practices in pediatric populations and the development of alternative strategies to mitigate risks. The diverse impacts of general anesthesia on CNS illustrate the necessity of a holistic amount of perioperative management. Understanding these mechanisms may help clinicians create strategies to minimize these neurologic and olfactory disturbances and possibly improve patient outcomes long-term [2].

## 4. Anatomy and Physiology of the Olfactory System

The olfactory system is a highly specialized sensory network that plays a critical role in detecting and processing odorants, contributing to essential functions like environmental awareness, flavor perception, and social interaction. Unlike other sensory modalities, the olfactory pathway has a direct and unique connection to the CNS. Odorant molecules are detected by olfactory receptor neurons (ORNs) located in the olfactory epithelium, a thin layer of tissue in the nasal cavity. This direct link to limbic structures highlights the olfactory system’s integration with memory, emotion, and cognition, making it particularly susceptible to neurological insults [32]. One of the defining features of the olfactory system is its remarkable regenerative capacity. Unlike most neurons in the CNS, ORNs undergo continuous turnover, with new neurons being generated from basal stem cells in the olfactory epithelium [33]. This regenerative ability is crucial for maintaining olfactory function, as ORNs are directly exposed to environmental toxins, pathogens, and other harmful agents. However, this regenerative process can be disrupted by systemic insults, including inflammation, ischemia, and neurotoxicity, all of which may occur during or after GA [3]. For instance, anesthetic agents, such as isoflurane and sevoflurane, have been associated with increased oxidative stress and apoptosis, which could impair neurogenesis in the olfactory epithelium and bulb [34]. The olfactory bulb, located at the base of the frontal lobe, is a key structure in the olfactory pathway and serves as a relay station for sensory information. It is densely connected to cortical and subcortical regions, including the orbitofrontal cortex and the limbic system, which are involved in higher-order processing of odors, memory integration, and emotional responses [35]. Importantly, the olfactory bulb is highly vascularized and relies on robust blood flow to maintain its function. Anesthetic-induced alterations in cerebral perfusion, as well as systemic hypoxia, can compromise the olfactory bulb’s ability to process sensory signals, leading to transient or persistent olfactory dysfunction [36]. Furthermore, the olfactory bulb is a site of neuroplasticity, where synaptic connections are constantly adjusted in response to sensory input. Disruptions in this plasticity, potentially caused by anesthesia-related neuroinflammation or metabolic changes, may further impair olfactory function [37]. The olfactory system is also uniquely vulnerable due to its exposure to environmental and systemic factors. Unlike other sensory systems, the olfactory neurons are in direct contact with the external environment, making them susceptible to damage from airborne toxins, viruses, and inflammatory agents. This exposure can create a pathway for systemic conditions, including those triggered by surgical stress and anesthesia, to directly affect the CNS. For example, studies have shown that systemic inflammation caused by surgical procedures can lead to neuroinflammation in the olfactory bulb, potentially contributing to postoperative olfactory dysfunction [38]. This interplay between systemic and neural factors underscores the olfactory system’s sensitivity to perioperative conditions. In addition to its role in sensory perception, the olfactory system is increasingly recognized as a potential biomarker for neurological health. Olfactory dysfunction is often an early symptom of neurodegenerative diseases, such as Parkinson’s disease and Alzheimer’s disease, preceding motor or cognitive symptoms by years [13]. The mechanisms underlying this association include neuroinflammation, synaptic loss, and the accumulation of pathological proteins, such as alpha-synuclein and amyloid-beta, in the olfactory bulb and cortex [25]. Interestingly, similar mechanisms have been observed in studies investigating the neurotoxic effects of anesthetic agents, suggesting a potential overlap between anesthesia-induced olfactory dysfunction and the early stages of neurodegeneration. Given these potential links between olfactory dysfunction, cognitive decline, and anesthesia-related complications, comprehensive preoperative assessment, as outlined in recent guidelines, becomes crucial for identifying patients at higher risk of postoperative neurological disturbances [1,39,40]. Anatomical and physiological differences across patient populations may also influence the vulnerability of the olfactory system to anesthesia-related disturbances. Age is a significant factor, as the regenerative capacity of the olfactory epithelium declines with age and the olfactory bulb undergoes structural and functional changes over time [41,42]. These age-related changes may explain why older adults are more likely to experience persistent olfactory dysfunction following anesthesia. Similarly, individuals with pre-existing conditions, such as chronic rhinosinusitis, diabetes, or neurodegenerative diseases, may have a reduced baseline olfactory function, making them more susceptible to further impairments after surgery [43]. The unique anatomical and physiological features of the olfactory system not only make it vulnerable to anesthesia-induced damage but also position it as a potential model for studying neuroplasticity and recovery following general anesthesia exposure.

## 5. Olfactory Dysfunction: Clinical Features and Etiology

Olfactory dysfunction encompasses a spectrum of impairments ranging from partial loss of smell (hyposmia) to complete loss (anosmia), as well as qualitative alterations, such as distorted smell perception (parosmia) or the perception of odors that are not present (phantosmia) [44]. These disturbances can have profound effects on quality of life, impairing basic functions like detecting hazards (e.g., smoke or spoiled food) and reducing the enjoyment of eating and social interactions. In the context of GA, olfactory dysfunction is a relatively underexplored phenomenon, yet it may be an important marker of broader neurological or systemic effects resulting from surgery and anesthetic exposure. The etiology of olfactory dysfunction is multifactorial, with causes ranging from local damage to the olfactory epithelium to broader neurological or systemic conditions. Localized causes include sinonasal diseases, such as chronic rhinosinusitis and nasal polyps, which obstruct the olfactory cleft and impair odor detection [43]. In the context of general anesthesia, the etiology of olfactory dysfunction is likely multifactorial, involving both direct effects on the olfactory epithelium and broader neurological impacts. This complexity underscores the need for comprehensive pre- and postoperative olfactory assessments, particularly in high-risk populations, to better understand and manage this often-overlooked complication. However, neurological causes, including CNS damage, neuroinflammation, and neurodegenerative diseases, play an equally significant role in olfactory impairment. For instance, the olfactory bulb and cortex are among the first regions affected in neurodegenerative disorders like Alzheimer’s disease and Parkinson’s disease, making olfactory dysfunction a potential early biomarker of these conditions [12]. Similarly, exposure to toxins, infections, or systemic inflammatory responses, such as those triggered by surgery and anesthesia, can disrupt the delicate balance of the olfactory system [44]. Olfactory dysfunction following GA likely results from a combination of systemic and localized mechanisms. Systemically, the inflammatory response induced by surgery and anesthetic agents plays a central role. Studies have shown that anesthetic agents, such as isoflurane and sevoflurane, can activate microglia and astrocytes, leading to the release of pro-inflammatory cytokines in the CNS [45]. This neuroinflammatory response can impair the function of olfactory pathways, particularly the olfactory bulb, which is highly sensitive to inflammatory and oxidative stress [46]. Additionally, systemic hypoxia or ischemia caused by fluctuations in cerebral blood flow during anesthesia may affect the olfactory bulb and cortex, leading to transient or persistent olfactory dysfunction [47]. Localized mechanisms of olfactory dysfunction during or after anesthesia include damage to the olfactory epithelium. The olfactory receptor neurons (ORNs) within the epithelium are exposed to direct environmental insults, such as desiccation or chemical irritation from intubation or anesthetic gases [40,48]. Studies have demonstrated that oxidative stress induced by volatile anesthetics can impair neurogenesis, potentially compromising the renewal of olfactory neurons and leading to functional deficits [34]. These findings suggest that the effects of anesthesia on the olfactory system are not limited to the CNS but also involve peripheral structures. However, in some cases, dysfunction may persist, particularly in individuals with pre-existing vulnerabilities. Risk factors for prolonged olfactory dysfunction include advanced age, pre-existing sinonasal or neurological conditions, and prolonged exposure to anesthetic agents [49]. Age-related declines in olfactory function, known as presbyosmia, are particularly relevant, as older adults already experience reduced neurogenesis and structural degeneration of the olfactory bulb and cortex [50]. These age-related changes may exacerbate the impact of anesthesia-induced neurotoxicity, leading to more pronounced and persistent olfactory impairments. Another important consideration is the relationship between olfactory dysfunction and postoperative cognitive decline (POCD). Both conditions share common mechanisms, including neuroinflammation, oxidative stress, and hypoxic damage, suggesting that olfactory dysfunction may serve as a clinical marker for broader neurological complications following surgery [51]. For instance, studies have reported that patients with postoperative cognitive impairment often exhibit concurrent deficits in olfactory function, potentially reflecting shared damage to the hippocampus, the orbitofrontal cortex, and related neural circuits [41]. This overlap underscores the need for comprehensive perioperative assessments that include both cognitive and olfactory evaluations. Environmental and genetic factors also contribute to the variability in olfactory dysfunction outcomes. Environmental exposures, such as smoking or chronic exposure to air pollutants, can damage the olfactory epithelium and reduce baseline olfactory function [45]. Genetic factors, particularly variations in olfactory receptor genes, have been associated with differences in olfactory function and may influence individual susceptibility to olfactory impairments [52]. Identifying these risk factors could help stratify patients based on their vulnerability and guide personalized anesthetic strategies to reduce complications.

## 6. Neurological and Olfactory Disturbances After General Anesthesia

**Figure 1:**
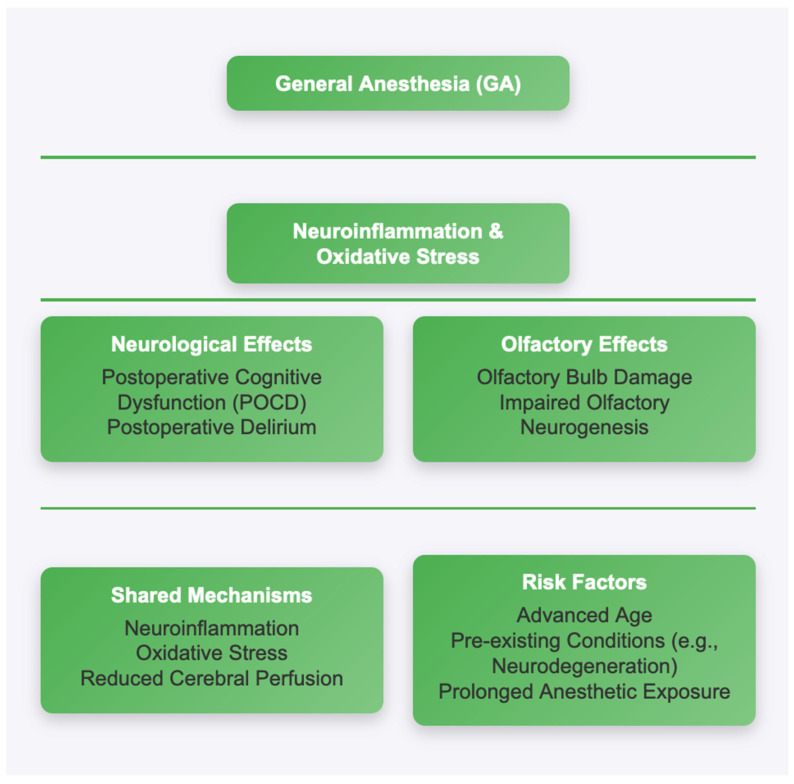
Flow diagram of mechanisms.

The potential interplay between neurological and olfactory disturbances following GA has garnered increasing attention in recent years [53]. These overlapping pathways raise the possibility that olfactory dysfunction could serve as an early indicator of broader neurological impairments after surgery, particularly in vulnerable populations, such as the elderly or those with pre-existing neurological conditions. POCD is characterized by impairments in memory, attention, and executive function that can persist for weeks or months after surgery, particularly in older adults [7,8]. Both conditions have been linked to anesthesia-induced neuroinflammation, which involves the activation of microglia and the release of pro-inflammatory cytokines, such as interleukin-6 (IL-6) and tumor necrosis factor-alpha (TNF-α) [21]. These inflammatory processes can disrupt synaptic function and neurogenesis in brain regions critical for both cognition and sensory processing, including the hippocampus, the prefrontal cortex, and the olfactory bulb [14]. Olfactory dysfunction, though less frequently studied in the context of GA, may be a related outcome of these same mechanisms. The olfactory bulb, as an extension of the central nervous system, is particularly vulnerable to systemic and neural insults, including the neuroinflammatory and oxidative stress pathways triggered by anesthetic agents [36]. For example, volatile anesthetics, such as isoflurane and sevoflurane, have been shown to impair olfactory neurogenesis and increase apoptosis in the olfactory bulb and the epithelium, potentially leading to transient or persistent olfactory deficits [35]. Furthermore, disruptions in cerebral perfusion during anesthesia, particularly in older patients or those with pre-existing vascular conditions, may compromise the delicate vasculature of the olfactory bulb and cortex, exacerbating sensory dysfunction [11]. One key area of overlap between neurological and olfactory disturbances is the role of oxidative stress. Anesthetic agents are known to increase the production of reactive oxygen species (ROS), which can damage cellular structures and impair mitochondrial function in neurons [9]. In the olfactory system, oxidative stress can disrupt the regeneration of olfactory receptor neurons (ORNs) and impair the synaptic plasticity of the olfactory bulb, leading to functional deficits [5]. Similar mechanisms have been implicated in the development of POCD, where oxidative damage to the hippocampus and the prefrontal cortex contributes to memory and attention deficits [22] (Figure 1).

This shared vulnerability underscores the interconnectedness of neurological and olfactory impairments in the postoperative period. Another potential link between olfactory and cognitive disturbances is their shared relationship with aging and neurodegeneration. Age-related declines in olfactory function, known as presbyosmia, are common and reflect structural and functional changes in the olfactory bulb and the cortex, including reduced neurogenesis and synaptic density [54]. Interestingly, anesthetic agents have been shown to exacerbate pathological processes associated with neurodegeneration, such as amyloid-beta aggregation and tau hyperphosphorylation, raising concerns about their potential long-term effects on brain health [40]. For example, studies have demonstrated that exposure to isoflurane can accelerate amyloid-beta deposition in animal models, potentially linking anesthesia-induced olfactory and cognitive impairments to early neurodegenerative changes [40]. The temporal relationship between neurological and olfactory disturbances remains an area of active research. In some cases, olfactory dysfunction appears to precede cognitive impairments, suggesting that it may serve as an early biomarker for anesthesia-induced neurological damage [35]. However, it is also possible that these disturbances occur simultaneously or that cognitive impairments exacerbate sensory deficits through disruptions to higher-order olfactory processing. For instance, the orbitofrontal cortex, a region involved in both olfactory and cognitive functions, is frequently affected in patients with POCD, potentially contributing to co-occurring sensory and cognitive impairments [41]. Risk factors for the development of olfactory and neurological disturbances after GA include advanced age, pre-existing cognitive or sensory deficits, and comorbidities, such as diabetes, cardiovascular disease, or chronic inflammation [38]. Patients with these risk factors are more likely to experience exaggerated neuroinflammatory and oxidative responses to anesthesia, increasing their susceptibility to both cognitive and sensory impairments. Additionally, the type and duration of anesthetic exposure may play a role; for example, prolonged surgeries or the use of certain volatile anesthetics have been associated with greater risks of postoperative complications [16]. The interplay between neurological and olfactory disturbances following GA highlights the need for a more integrated approach to perioperative care. Both conditions share common mechanisms, including neuroinflammation, oxidative stress, and vascular dysfunction, suggesting that they may arise from similar underlying processes. Recognizing olfactory dysfunction as a potential early marker of broader neurological impairments could provide valuable insights into patient outcomes and guide the development of targeted interventions to reduce postoperative complications. The interplay between neurological and olfactory disturbances following general anesthesia necessitates an integrated approach to perioperative care. By recognizing olfactory dysfunction as a potential early marker of broader neurological impairments, clinicians can develop more comprehensive assessment protocols and targeted interventions. Future research should focus on elucidating the temporal and mechanistic relationships between these disturbances, potentially leading to improved predictive models and preventive strategies.

## 7. Risk Factors and Vulnerable Populations

The risk of developing neurological and olfactory disturbances after GA is influenced by a variety of factors, including age, pre-existing health conditions, genetic predispositions, and perioperative variables. These risk factors not only determine the likelihood of postoperative complications but also influence the severity and duration of impairments. Understanding these vulnerabilities is crucial for identifying high-risk patients and tailoring anesthetic and surgical strategies to minimize adverse outcomes. Age is one of the most significant risk factors for both neurological and olfactory dysfunction after GA. Older adults are particularly vulnerable to POCD and olfactory disturbances due to age-related declines in neuronal plasticity, vascular function, and immune regulation [20]. Structural changes in the brain, such as hippocampal atrophy and reduced neurogenesis in the olfactory bulb, further exacerbate the impact of anesthesia on cognitive and sensory functions [20]. For instance, studies have shown that older adults experience prolonged recovery of olfactory function following anesthesia, with some individuals exhibiting persistent anosmia or hyposmia [55]. Similarly, the incidence of POCD is significantly higher in elderly surgical patients, with cognitive impairments potentially lasting for months or longer [56]. Pediatric populations, while less studied, also represent a vulnerable group. The developing brain is highly sensitive to disruptions in neural activity, making children more susceptible to the neurotoxic effects of anesthetic agents. Animal studies have demonstrated that exposure to GA during critical periods of brain development can lead to widespread neuronal apoptosis and long-term deficits in cognitive and sensory function [7]. Clinically, prolonged or repeated exposure to anesthetics in early childhood has been associated with impaired neurodevelopmental outcomes, including deficits in memory, attention, and language skills [30]. While the effects of anesthesia on the olfactory system in children remain poorly understood, the overlap between sensory and cognitive pathways suggests that similar vulnerabilities may exist (Table 1).

Pre-existing medical conditions also play a critical role in determining the risk of postoperative complications. Patients with neurodegenerative diseases, such as Alzheimer’s or Parkinson’s disease, are particularly susceptible to anesthesia-induced exacerbation of cognitive and sensory dysfunctions [31]. Similarly, individuals with chronic conditions, such as diabetes, cardiovascular disease, or chronic obstructive pulmonary disease (COPD), are at increased risk due to systemic inflammation, vascular dysfunction, and reduced baseline neural resilience [22]. These comorbidities can amplify the neuroinflammatory and oxidative stress responses triggered by anesthesia, leading to more severe and prolonged impairments. Genetic predispositions may also influence susceptibility to anesthesia-related neurological and olfactory dysfunction. Variations in genes associated with inflammation, oxidative stress, and neuroplasticity have been implicated in differential responses to anesthetic exposure. For example, polymorphisms in the apolipoprotein E (APOE) gene, particularly the APOE-ε4 allele, have been linked to an increased risk of POCD and neurodegeneration after surgery [53]. Similarly, genetic variations that affect the regenerative capacity of the olfactory epithelium or the integrity of the blood–brain barrier may contribute to individual differences in the severity of olfactory dysfunction post anesthesia [57]. Identifying these genetic markers could pave the way for personalized anesthetic strategies that minimize risks in genetically predisposed individuals. Perioperative factors, including the type and duration of anesthesia, also significantly influence outcomes. Volatile anesthetics, such as isoflurane and sevoflurane, have been associated with higher levels of neuroinflammation and oxidative stress compared to intravenous agents like propofol [55]. Prolonged exposure to anesthetic agents increases the risk of cumulative neurotoxic effects, particularly in lengthy or complex surgeries [42]. Additionally, intraoperative factors, such as hypoxia, hypotension, and fluctuations in cerebral perfusion, can exacerbate neural and sensory damage, particularly in patients with pre-existing vascular or respiratory conditions [35]. Postoperative factors, including inadequate pain management and prolonged sedation, may also contribute to cognitive and sensory impairments by disrupting sleep, increasing stress, and prolonging systemic inflammation [54]. Environmental and lifestyle factors further modify the risk of postoperative complications. Chronic exposure to environmental toxins, such as air pollution or smoking, can impair baseline olfactory function and reduce neural resilience to anesthesia-induced damage [26]. Similarly, poor nutritional status, lack of physical activity, and other lifestyle factors that affect systemic inflammation and vascular health may increase susceptibility to cognitive and sensory dysfunctions [45]. Understanding the complex interplay of risk factors for post-anesthetic neurological and olfactory disturbances is crucial for developing personalized anesthetic protocols.

## 8. Preventive Strategies and Management

Given the potential for neurological and olfactory disturbances after GA, preventive strategies and effective management are critical for minimizing postoperative complications and improving patient outcomes. These strategies involve a combination of preoperative risk assessment, intraoperative optimization, and postoperative care, with a focus on reducing neuroinflammation, oxidative stress, and vascular dysfunction. Tailoring interventions to individual patient profiles, particularly in high-risk populations, is essential for achieving the best results.

In addition to anesthetic selection, there are perioperative strategies that can further increase patient safety and decrease the risk of cognitive and sensory complications. Preventing ischemia to cognitive and olfactory centers can be achieved through careful intracranial blood pressure management, which we refer to as fluid therapy, as well as cerebral oximetry monitoring to optimize cerebral perfusion and oxygenation during surgery. The same can be said for limiting exposure to prolonged anesthesia and avoiding sedation in the early postoperative period, as this potentially facilitates cognitive recovery.

Preoperative risk assessment is the cornerstone of preventive strategies. Identifying patients at higher risk, such as the elderly, children, or individuals with comorbidities, like neurodegenerative diseases or diabetes, allows clinicians to implement targeted interventions. Comprehensive preoperative evaluations should include assessments of cognitive function, olfactory performance, and comorbid conditions that may exacerbate postoperative complications [39].

Several validated cognitive and olfactory assessments are available to guide preoperative risk stratification. The most common cognitive screening tests are the Mini-Mental State Diagnostic (MMSE) and the Montreal Cognitive Assessment (MoCA), which can rapidly screen for potential cognitive impairment. A more detailed assessment can be useful, particularly in older patients, e.g., the Clock Drawing Test or the Trail Making Test to reflect executive function and visuospatial skills. Olfactory evaluation can be performed by using the University of Pennsylvania Smell Identification Test (UPSIT) or the shorter Sniffin’ Sticks test, both of which provide quantitative measures of olfactory function. These tests can be reasonably quick to administer and can identify relatively subtle olfactory deficits that may be an indicator of increased susceptibility to postoperative adverse events.

Baseline cognitive testing or olfactory assessments could help identify subtle impairments that might predispose patients to postoperative dysfunction. Recent guidelines from the European Society of Anesthesiology and Intensive Care Medicine (ESAIC) on postoperative delirium in adult patients provide valuable, evidence-based recommendations for prevention and management. These guidelines emphasize the importance of multicomponent non-pharmacological interventions, including early mobilization, sleep hygiene, and cognitive stimulation. They also highlight the role of depth of anesthesia monitoring and the judicious use of certain medications.

**Figure 2:**
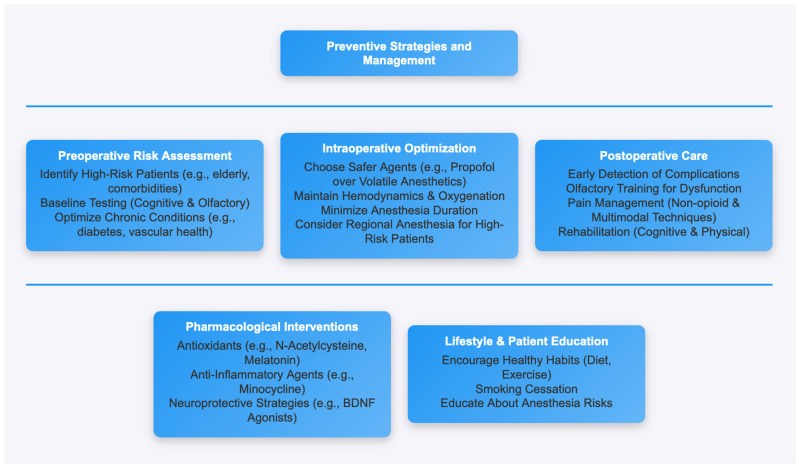
Flow diagram of preventive strategies available.

Patient-centered anesthetic management is important to avoid anesthesia-related complications. Selection of anesthetic agents, depth of anesthesia, and adjunctive medications must be tailored to each patient’s risk profile. The use of volatile anesthetics, including isoflurane and sevoflurane, with increased neuroinflammation, oxidative stress, and amyloid-beta accumulation may lead to cognitive decline among older patients or those with other factors predisposing them to neurodegenerative disease. Intravenous anesthetics (e.g., propofol and dexmedetomidine), in contrast, have demonstrated neuroprotective effects through oxidative stress reduction and neuroinflammatory pathway modulation. Indeed, dexmedetomidine, via its sedative and anti-inflammatory properties, has been shown to reduce the incidence of postoperative delirium and POCD in humans. With the exception of previous studies showing the efficacy of regional anesthetic in specific populations (e.g., the elderly or high-risk patients) as a suitable alternative to general anesthesia for selected procedures to avoid systemic neurotoxic effects of inhalational drugs, regional anesthesia has not routinely been used in outpatient settings. Multimodal anesthesia approaches have also been suggested to reduce total neurotoxicity, as they include lower doses of various agents while still achieving sufficient anesthesia depth. Additionally, optimizing the management of chronic conditions, such as controlling blood glucose levels in diabetic patients or ensuring adequate treatment for vascular diseases, may reduce the systemic stress associated with surgery and anesthesia [20]. The choice of anesthetic agents and techniques plays a significant role in minimizing postoperative complications. Volatile anesthetics, such as isoflurane and sevoflurane, have been associated with higher levels of neuroinflammation and oxidative stress compared to intravenous agents like propofol [58] (Figure 2). The integration of neuroprotective pharmacological agents along with personalized anesthesia appears to hold tremendous potential in the future. Various antioxidants, including N-acetylcysteine and melatonin, have been studied for their potential to reduce the oxidative stress and neuroinflammation linked with anesthetic exposure. Minocycline is also an anti-inflammatory agent that has been shown to be neuroprotective by reducing microglial activation and neuronal apoptosis. Future studies should investigate these and other interventions in the context of a holistic approach to prevent cognitive and sensory decline in the wake of anesthesia.

Propofol has demonstrated neuroprotective effects in certain contexts, including reduced mitochondrial damage and lower levels of reactive oxygen species (ROS) production [42]. Implementing multimodal anesthetic strategies, which combine lower doses of different agents, can also reduce the overall neurotoxic burden. Additionally, regional anesthesia may be considered as an alternative to GA in certain surgical procedures, particularly in high-risk populations, as it avoids the systemic effects of general anesthetic agents [28]. Intraoperative monitoring and optimization are essential for preventing complications. Maintaining stable hemodynamics, adequate oxygenation, and optimal cerebral perfusion reduces the risk of hypoxia and ischemia, which can exacerbate both neurological and olfactory impairments [43].

Intraoperative monitoring and optimization are also important to avoid complications. Stable hemodynamics, adequate oxygenation, and optimal cerebral perfusion are critical components of therapeutic care aimed at reducing the risk of hypoxia and ischemia, which can worsen neurological [23] and olfactory [35] deficits. Cerebral oximetry is also an advanced monitoring technique that can help ensure profound blood flow in the brain and the olfactory bulb [23]. Furthermore, reducing the length of anesthesia exposure and unnecessary deep sedation could decrease cumulative neurotoxic effects of anesthetic agents [59]. In the elderly, titration of anesthetic doses according to age-related pharmacodynamic changes is important to prevent excessive exposure [35]. Multimodal anesthetic strategies, which rely on lower doses of several co-administered agents, i.e., adjunct therapy in the form of a neurotoxicant pair (MR, title), may also reduce the total neurotoxic burden. Moreover, in many surgical procedures, regional anesthesia could be regarded as an alternative to GA, specifically in high-risk populations, considering the avoidance of systemic side effects of general anesthetic agents [28]. Postoperative care focuses on early identification and management of complications to promote recovery. Regular cognitive and sensory evaluations in the immediate postoperative period can help detect subtle impairments in their early stages, allowing for timely intervention [54]. For patients experiencing olfactory dysfunction, treatments like olfactory training—a structured regimen of repeated exposure to specific odors—have shown promise in enhancing recovery and improving olfactory function [54]. This approach leverages the neuroplasticity of the olfactory system and may be particularly effective in cases of transient dysfunction caused by anesthesia-induced damage. Management of POCD and delirium involves addressing contributing factors, such as pain, sleep disturbances, and systemic inflammation. Multimodal analgesia, which combines non-opioid medications with regional anesthesia techniques, can provide effective pain relief while minimizing the cognitive side effects of opioids [60]. Ensuring adequate sleep hygiene and promoting early mobilization may also help reduce the risk of delirium and facilitate cognitive recovery. For patients with persistent POCD, cognitive rehabilitation programs that focus on memory, attention, and executive function may aid in long-term recovery [8]. Pharmacological interventions to mitigate the neurotoxic effects of anesthetic agents are an area of active research. Antioxidants, such as N-acetylcysteine and melatonin, have shown promise in animal studies for reducing oxidative stress and preserving neuronal function after anesthesia exposure [7]. Similarly, anti-inflammatory agents that target microglial activation, such as minocycline, may help reduce neuroinflammation and protect against cognitive and sensory impairments [21]. While these approaches are not yet widely adopted in clinical practice, they represent potential future strategies for improving patient outcomes. Lifestyle modifications and patient education are also important components of long-term management. Encouraging healthy behaviors, such as regular physical activity, a balanced diet rich in antioxidants, and smoking cessation, can improve overall neural resilience and reduce the risk of persistent postoperative impairments [21]. Educating patients about the potential risks of anesthesia, particularly for those with pre-existing vulnerabilities, allows for more informed decision making and fosters better collaboration between patients and healthcare providers [61]. The development of effective preventive strategies requires a multidisciplinary approach integrating insights from anesthesiology, neurology, and olfactory science.

## 9. Future Directions and Research Opportunities

Our findings may serve as a guide for a precision medicine approach in the field of anesthesia that can help improve patient outcomes and decrease long-term morbidity. Future studies should target biomarkers of anesthesia-induced neurological and sensory injury, targeted neuroprotective therapies, and improved perioperative management protocols for high-risk populations. The next step should be ensuring patient safety, which would involve cardioprotective and neuroprotective anesthetic protocols tailored to each patient derived from integrating their genetic predispositions, baseline cognitive status, and comorbidities before surgery.

The growing recognition of neurological and olfactory disturbances as potential complications of GA underscores the need for further research to better understand their mechanisms, risk factors, and management strategies. While significant progress has been made in identifying the neuroinflammatory and oxidative pathways involved in these impairments, many questions remain unanswered, particularly regarding the long-term effects of anesthesia exposure and the interplay between sensory and cognitive dysfunction. Future studies should aim to address these gaps and develop innovative approaches to minimize postoperative complications. One key area for future research is the development of biomarkers to predict and monitor neurological and olfactory dysfunction following GA. Biomarkers, such as inflammatory cytokines (e.g., interleukin-6, tumor necrosis factor-alpha) and markers of oxidative stress (e.g., malondialdehyde, reactive oxygen species), could help identify patients at risk of postoperative complications and track their recovery trajectories [20]. Additionally, imaging techniques, such as functional MRI and PET scans, may provide insights into structural and functional changes in the brain and the olfactory system during and after anesthesia exposure [42]. Identifying reliable and non-invasive biomarkers could facilitate early intervention and improve individualized patient care. Another promising avenue is the investigation of anesthetic agents and their differential effects on the nervous system. While volatile anesthetics, such as isoflurane and sevoflurane, have been extensively studied, further research is needed to explore the neuroprotective potential of alternative agents, like dexmedetomidine and ketamine [35]. Dexmedetomidine, for instance, has shown anti-inflammatory and neuroprotective properties in both animal and clinical studies, making it a potential candidate for reducing anesthesia-induced neuroinflammation and sensory impairments [62]. Similarly, low-dose ketamine, when used as part of multimodal anesthesia, may offer protective effects against neurocognitive decline due to its NMDA receptor antagonism and anti-inflammatory properties [63]. Comparative studies evaluating the long-term outcomes associated with different anesthetic regimens could lead to safer and more effective protocols. The role of neurogenesis and plasticity in recovery from anesthesia-induced damage is another critical area of exploration. The olfactory system, with its unique capacity for continuous neurogenesis, provides a valuable model for studying the regenerative potential of the nervous system following anesthetic exposure [64]. Understanding the factors that influence neural regeneration, such as the impact of age, inflammation, and oxidative stress, could inform strategies to enhance recovery in both the olfactory and cognitive domains. For example, interventions that promote neurogenesis, such as exercise, enriched environments, and pharmacological agents, like brain-derived neurotrophic factor (BDNF) agonists, may hold promise for mitigating long-term impairments [5]. The long-term effects of anesthesia exposure on vulnerable populations, such as children and the elderly, represent a critical knowledge gap. While short-term studies have demonstrated cognitive and sensory impairments following anesthesia, longitudinal research is needed to assess whether these effects persist or contribute to neurodegenerative processes later in life [65]. For pediatric populations, studies should focus on the impact of repeated or prolonged anesthesia exposure on neurodevelopmental outcomes, as well as potential protective strategies to mitigate risks during critical periods of brain development [20]. In elderly patients, research should investigate whether anesthesia accelerates age-related declines in cognitive and olfactory function or interacts with pre-existing neurodegenerative conditions. Future studies should explore how these comorbidities influence neuroinflammatory and oxidative stress responses to anesthesia and whether targeted interventions, such as anti-inflammatory therapies or vascular support, can improve outcomes in these populations [66]. The potential for pharmacological interventions to prevent or treat anesthesia-related complications represents an exciting frontier. Antioxidants, such as N-acetylcysteine, melatonin, and edaravone, have shown promise in preclinical models for reducing oxidative stress and preserving neural function [67]. Similarly, anti-inflammatory agents like minocycline and non-steroidal anti-inflammatory drugs (NSAIDs) have demonstrated efficacy in reducing microglial activation and neuroinflammation in animal studies [68]. Clinical trials are needed to evaluate the safety and efficacy of these agents in preventing or mitigating neurological and olfactory impairments in surgical patients. Additionally, the use of neuroprotective strategies, such as perioperative administration of BDNF agonists or NMDA receptor modulators, could provide new therapeutic options [69]. Finally, the integration of technology and personalized medicine into perioperative care has the potential to revolutionize the prevention and management of postoperative complications. Advances in machine learning and predictive analytics could help identify at-risk patients based on their genetic, physiological, and clinical profiles, enabling tailored anesthetic protocols and monitoring strategies [14]. Wearable devices and remote monitoring systems could also play a role in tracking cognitive and sensory function in the postoperative period, providing real-time feedback to clinicians and facilitating early intervention [70,71].

## 10. Conclusions

Future research should focus on understanding the complex mechanisms underlying neurological and olfactory disturbances after GA, with an emphasis on identifying biomarkers, exploring alternative anesthetic agents, and developing targeted interventions. Longitudinal studies in vulnerable populations, such as children and the elderly, are particularly important for assessing the long-term effects of anesthesia on brain health. By leveraging advances in neuroscience, pharmacology, and technology, researchers and clinicians can work toward reducing the burden of postoperative complications and improving patient outcomes.

## Figures and Tables

**Table 1:** Risk factors and vulnerable populations for olfactory disorders.

Risk Factor/Vulnerable Population	Description	Associated Complications	Key References
Advanced Age	Reduced neuronal plasticity, vascular function, and immune regulation	Higher incidence of POCD, prolonged recovery of olfactory function	[20,55,56]
Pediatric Populations	Developing brain sensitive to neurotoxic effects	Potential long-term cognitive and sensory deficits	[7,30]
Neurodegenerative Diseases	Pre-existing conditions like Alzheimer’s or Parkinson’s	Exacerbation of cognitive and sensory dysfunctions	[31,41]
Chronic Conditions	Diabetes, cardiovascular disease, COPD	Increased risk due to systemic inflammation and vascular dysfunction	[22]
Genetic Predispositions	e.g., APOE-ε4 allele	Increased risk of POCD and neurodegeneration	[53,57]
Prolonged Anesthesia Exposure	Extended duration of surgery and anesthesia	Higher risk of cumulative neurotoxic effects	[42,55]
Environmental Factors	Chronic exposure to toxins, smoking	Impaired baseline olfactory function, reduced neural resilience	[26,45]

## Data Availability

No new data were created or analyzed in this study.

## Abbreviations

The following abbreviations are used in this manuscript:

GA	General anesthesia
NSAID	Non-steroidal anti-inflammatory drug
POCD	Postoperative cognitive dysfunction
GABA	Gamma-aminobutyric acid
CNS	Central nervous system
ORN	Olfactory receptor neuron
ROS	Reactive oxygen species
TNF-α	Tumor necrosis factor-Alpha
IL-6	Interleukin 6
APO-E	Apolipoprotein E
NMDA	N-methyl-D-aspartate
